# Supplementary material for: Developmental Vitamin D Deficiency in Pregnant Rats Does Not Induce Preeclampsia
Source: Nutrients. 2021 Nov 26;13(12):4254. doi: 10.3390/nu13124254 (PMC8707812; doi:10.3390/nu13124254)
Supplement: Supplementary file 1 [file nutrients-13-04254-s001.zip › nutrients-1457301-supplementary.pdf]

Supplementary data

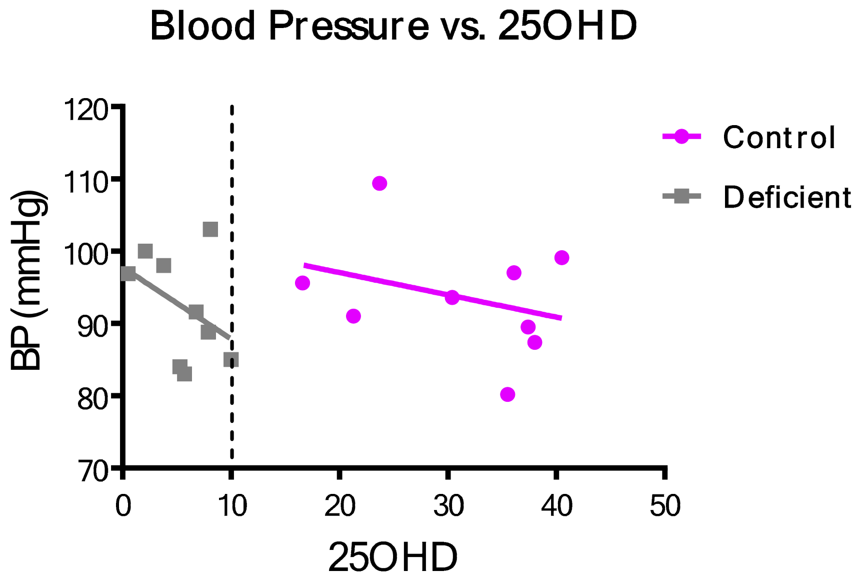

Figure S1. Pairwise correlations between maternal blood pressure and 25OHD levels at gestational day 19. There was no statistically significant correlation between blood pressure and 25OHD levels in control r = 0.10, p = 0.39 or deficient r = 0.16, p = 0.28 dams. BP=blood pressure, 25OHD = 25-hydroxyvitamin D. n= 9 control dams, n= 9 vitamin D deficient dams.
